# Supplementary material for: Overlapping cell population expression profiling and regulatory inference in C. elegans
Source: BMC Genomics. 2016 Feb 29;17:159. doi: 10.1186/s12864-016-2482-z (PMC4772325; doi:10.1186/s12864-016-2482-z)
Supplement: Additional file 13: — Web supplement. (DOC 21 kb) [file 12864_2016_2482_MOESM13_ESM.zip › sortWeb/clusters/hier.300.clusters/29.html]

Cluster 29 

## Cluster 29

### Expression

| cnd-1 rep. 1 | cnd-1 rep. 2 | cnd-1 rep. 3 | pha-4 rep. 1 | pha-4 rep. 2 | pha-4 rep. 3 | ceh-27 | ceh-36 | ceh-6 | F21D5.9 | mir-57 | mls-2 | pal-1 | pros-1 | ttx-3 | unc-130 | hlh-16 | irx-1 | ceh-6 (+) hlh-16 (+) | ceh-6 (+) hlh-16 (-) | ceh-6 (-) hlh-16 (+) | cnd-1 singlets | pha-4 singlets | 0 | 60 | 120 | 150 | 180 | 240 | 330 | 390 | 420 | 480 | 540 | 570 | 600 | 630 | 660 | NAME | Functional description |
| --- | --- | --- | --- | --- | --- | --- | --- | --- | --- | --- | --- | --- | --- | --- | --- | --- | --- | --- | --- | --- | --- | --- | --- | --- | --- | --- | --- | --- | --- | --- | --- | --- | --- | --- | --- | --- | --- | --- | --- |
|  |  |  |  |  |  |  |  |  |  |  |  |  |  |  |  |  |  |  |  |  |  |  |  |  |  |  |  |  |  |  |  |  |  |  |  |  |  | B0454.13 |  |
|  |  |  |  |  |  |  |  |  |  |  |  |  |  |  |  |  |  |  |  |  |  |  |  |  |  |  |  |  |  |  |  |  |  |  |  |  |  | F20E11.7 |  |
|  |  |  |  |  |  |  |  |  |  |  |  |  |  |  |  |  |  |  |  |  |  |  |  |  |  |  |  |  |  |  |  |  |  |  |  |  |  | C55A6.11 |  |
|  |  |  |  |  |  |  |  |  |  |  |  |  |  |  |  |  |  |  |  |  |  |  |  |  |  |  |  |  |  |  |  |  |  |  |  |  |  | Y26D4A.17 |  |
|  |  |  |  |  |  |  |  |  |  |  |  |  |  |  |  |  |  |  |  |  |  |  |  |  |  |  |  |  |  |  |  |  |  |  |  |  |  | C28A5.t1 |  |
|  |  |  |  |  |  |  |  |  |  |  |  |  |  |  |  |  |  |  |  |  |  |  |  |  |  |  |  |  |  |  |  |  |  |  |  |  |  | T07F8.1 |  |
|  |  |  |  |  |  |  |  |  |  |  |  |  |  |  |  |  |  |  |  |  |  |  |  |  |  |  |  |  |  |  |  |  |  |  |  |  |  | T19D7.9 |  |
|  |  |  |  |  |  |  |  |  |  |  |  |  |  |  |  |  |  |  |  |  |  |  |  |  |  |  |  |  |  |  |  |  |  |  |  |  |  | F42G2.5 |  |
|  |  |  |  |  |  |  |  |  |  |  |  |  |  |  |  |  |  |  |  |  |  |  |  |  |  |  |  |  |  |  |  |  |  |  |  |  |  | F40G12.10 |  |
|  |  |  |  |  |  |  |  |  |  |  |  |  |  |  |  |  |  |  |  |  |  |  |  |  |  |  |  |  |  |  |  |  |  |  |  |  |  | C47E8.10 |  |
|  |  |  |  |  |  |  |  |  |  |  |  |  |  |  |  |  |  |  |  |  |  |  |  |  |  |  |  |  |  |  |  |  |  |  |  |  |  | *set-28* | SET (trithorax/polycomb) domain containing |
|  |  |  |  |  |  |  |  |  |  |  |  |  |  |  |  |  |  |  |  |  |  |  |  |  |  |  |  |  |  |  |  |  |  |  |  |  |  | ZK930.5 |  |
|  |  |  |  |  |  |  |  |  |  |  |  |  |  |  |  |  |  |  |  |  |  |  |  |  |  |  |  |  |  |  |  |  |  |  |  |  |  | *acd-4* | ACid-sensitive Degenerin |
|  |  |  |  |  |  |  |  |  |  |  |  |  |  |  |  |  |  |  |  |  |  |  |  |  |  |  |  |  |  |  |  |  |  |  |  |  |  | *anr-57* | Antisense Non-coding RNA |
|  |  |  |  |  |  |  |  |  |  |  |  |  |  |  |  |  |  |  |  |  |  |  |  |  |  |  |  |  |  |  |  |  |  |  |  |  |  | ZK354.7 |  |
|  |  |  |  |  |  |  |  |  |  |  |  |  |  |  |  |  |  |  |  |  |  |  |  |  |  |  |  |  |  |  |  |  |  |  |  |  |  | C54D2.18 |  |
|  |  |  |  |  |  |  |  |  |  |  |  |  |  |  |  |  |  |  |  |  |  |  |  |  |  |  |  |  |  |  |  |  |  |  |  |  |  | F55G7.3 |  |
|  |  |  |  |  |  |  |  |  |  |  |  |  |  |  |  |  |  |  |  |  |  |  |  |  |  |  |  |  |  |  |  |  |  |  |  |  |  | B0393.14 |  |
|  |  |  |  |  |  |  |  |  |  |  |  |  |  |  |  |  |  |  |  |  |  |  |  |  |  |  |  |  |  |  |  |  |  |  |  |  |  | ZC21.12 |  |
|  |  |  |  |  |  |  |  |  |  |  |  |  |  |  |  |  |  |  |  |  |  |  |  |  |  |  |  |  |  |  |  |  |  |  |  |  |  | F22B8.9 |  |

### Phenotypes enriched

none found

### Anatomy terms enriched

none found

### GO terms enriched

none found

### Expression clusters enriched

none found

### Motifs enriched

|  |  |  |  |  |  |
| --- | --- | --- | --- | --- | --- |
| **Motif** | **Logo** | **Possible orthologs** | **Number of motifs in cluster** | **Enrichment** | **FDR corrected p** |
| V$HFH8\_01 |  | let-381 | 16 | 1.96 | 0.030 |
| ECC-1\_ERALPHA\_HudsonAlpha |  | nhr-71 | 13 | 2.33 | 0.040 |
| pTH5111 |  | aha-1 | 18 | 1.68 | 0.041 |
| pTH6447 |  | ceh-19 | 15 | 2.02 | 0.042 |
| pTH9290 |  | ceh-18 | 16 | 1.88 | 0.047 |
| TFEB\_f1 |  | hlh-30 | 14 | 2.12 | 0.048 |
| V$FAC1\_01 |  | gei-8 | 14 | 2.12 | 0.049 |

### Correlated (and anti-correlated) transcription factors

|  |  |
| --- | --- |
| **Transcription factor** | **Correlation** |
| ttx-3 | 0.51 |
| ZK686.5 | 0.51 |
| nhr-272 | 0.48 |
| hlh-34 | 0.46 |
| nhr-169 | 0.45 |
| hlh-14 | 0.44 |
| hlh-27 | 0.44 |
| fkh-10 | 0.44 |
| end-3 | 0.42 |
| T20H4.2 | 0.41 |
| ceh-12 | 0.40 |
| Y37F4.6 | 0.39 |
| lsl-1 | 0.37 |
| ceh-37 | 0.37 |
| mls-2 | 0.36 |
| nhr-188 | 0.35 |
| nhr-39 | 0.35 |
| 2L52.1 | 0.35 |
| flh-2 | 0.35 |
| nhr-254 | 0.35 |
| ceh-6 | 0.34 |
| nhr-108 | 0.34 |
| F10E7.11 | 0.34 |
| mab-23 | 0.34 |
| tbx-33 | 0.34 |
| nhr-33 | -0.31 |
| nhr-247 | -0.33 |
| mex-6 | -0.33 |
| nhr-189 | -0.34 |
| nhr-11 | -0.34 |
| dhhc-12 | -0.35 |
| nhr-113 | -0.35 |
| nhr-208 | -0.35 |
| ceh-79 | -0.36 |
| nhr-231 | -0.37 |
| nhr-115 | -0.37 |
| dpr-1 | -0.38 |
| tbx-32 | -0.38 |
| tbx-40 | -0.38 |
| klf-1 | -0.38 |
| Y116A8C.19 | -0.38 |
| nhr-267 | -0.39 |
| Y60A9.3 | -0.41 |
| nhr-261 | -0.41 |
| nhr-89 | -0.44 |
| nhr-262 | -0.45 |
| frpr-9 | -0.47 |
| nhr-155 | -0.59 |
| hlh-29 | -0.62 |
| ZK177.3 | -0.74 |

### ChIP peaks enriched

none found
